# S*tellera chamaejasme* and its constituents induce cutaneous wound healing and anti-inflammatory activities

**DOI:** 10.1038/srep42490

**Published:** 2017-02-21

**Authors:** Myungsuk Kim, Hee Ju Lee, Ahmad Randy, Ji Ho Yun, Sang-Rok Oh, Chu Won Nho

**Affiliations:** 1 Natural Products Research Center, Korea Institute of Science and Technology, Gangneung, Republic of Korea; 2 Convergence Research Center for Smart Farm Solution, Korea Institute of Science and Technology, Gangneung, Republic of Korea; 3 Systems Biotechnology Research Center, Korea Institute of Science and Technology, Gangneung, Republic of Korea; 4 Department of Biological Chemistry, Korea, University of Science and Technology, Daejeon, Republic of Korea

## Abstract

*Stellera chamaejasme* L. (Thymelaeaceae) is a perennial herb that is widely used in traditional Chinese medicine to treat tumours, tuberculosis and psoriasis. *S. chamaejasme* extract (SCE) possesses anti-inflammatory, analgesic and wound healing activities; however, the effect of *S. chamaejasme* and its active compounds on cutaneous wound healing has not been investigated. We assessed full-thickness wounds of Sprague-Dawley (SD) rats and topically applied SCE for 2 weeks. *In vitro* studies were performed using HaCaT keratinocytes, Hs68 dermal fibroblasts and RAW 264.7 macrophages to determine cell viability (MTT assay), cell migration, collagen expression, nitric oxide (NO) production, prostaglandin E_2_ (PGE_2_) production, inflammatory cytokine expression and β-catenin activation. *In vivo*, wound size was reduced and epithelisation was improved in SCE-treated SD rats. *In vitro*, SCE and its active compounds induced keratinocyte migration by regulating the β-catenin, extracellular signal-regulated kinase and Akt signalling pathways. Furthermore, SCE and its active compounds increased mRNA expression of type I and III collagen in Hs68 fibroblasts. SCE and chamechromone inhibited NO and PGE_2_ release and mRNA expression of inflammatory mediators in RAW 264.7 macrophages. SCE enhances the motility of HaCaT keratinocytes and improves cutaneous wound healing in SD rats.

Cutaneous wound healing is a dynamic process involving intricate interactions among a variety of inflammatory cells, extracellular matrix (ECM) molecules, parenchymal cells and soluble mediators^1^. The wound healing process consists of three steps: inflammatory, proliferative and remodelling^2^. Representative mechanisms associated with cutaneous wound healing are the β-catenin, extracellular signal-regulated kinase (ERK) and Akt signalling pathways^3^,^4^,^5^.

β-catenin signalling pathways play pivotal roles in embryonic development, cell proliferation and cell migration^6^,^7^,^8^. The β-catenin-dependent canonical pathway and β-catenin-independent noncanonical pathways^9^, are both relevant to wound healing^2^. During the re-epithelisation stage of wound healing^10^, β-catenin is activated, modulates cell migration and proliferation, and induces fibromatosis in hyperplastic wounds^11^.

The ERK and Akt signalling pathways are associated with regulation of cutaneous wound healing^12^,^13^. Activation and initiation of ERK and Akt signaling pathways by growth factors such as fibroblast growth factor and epidermal growth factor may induce the motility of keratinocytes and re-epithelialisation in wounded skin^14^,^15^. A study reports that suppressing these factors may delay corneal wound healing, suggesting that the wound healing process may be accelerated by stimulating the β-catenin, ERK and Akt signalling pathways^5^,^10^.

The transforming growth factor (TGF)-β1 signalling pathway recruits and phosphorylates Smad2 and Smad3 through a complex of type I and type II receptors. Phosphorylated receptor-regulated Smads (Smad2 and Smad3) then form a heteromeric complex with coSmad (Smad4) and accumulate in the nucleus, where they act as transcription factors and participate in the regulation of TGF-β1-responsive gene expression^16^,^17^. Administration of exogenous TGF-β1 accelerates the wound healing process through the increased accumulation of ECM molecules, such as type I and III collagen^18^.

Nitric oxide (NO) is a highly reactive free radical and is an essential signalling factor in various physiological processes and cell types^19^. Cyclooxygenase (COX) is essential for the conversion of arachidonic acid into prostaglandin H_2_, a precursor of various biologically active mediators including thromboxane A2, prostacyclin and prostaglandin E2 (PGE_2_)^20^. Production and levels of nitric oxide synthase (NOS)-derived NO and COX-2-derived PGE_2_ were increased in a model of inflammatory response that is induced by proinflammatory mediators such as tumour necrosis factor (TNF)-α, interleukin (IL)-1β, and lipopolysaccharide (LPS)^21^. This induced inflammatory response may trigger further damage to adjacent cells and tissues around the wound site, thus delaying the wound healing process^22^. Previous studies have reported that suppression of NO and PGE_2_ production by treatment with NOS and COX-2 inhibitors may prevent some types of injury^23^,^24^.

*Stellera chamaejasme* L. (Thymelaeaceae) is a famous toxic herb that is widely distributed in China, Mongolia, Russia and Korea. This herb has both toxic and therapeutic effects. It has been used as a pesticide and as a remedy for stubborn skin ulcers^25^ with antiviral^26^,^27^, antitumour^28^, antibacterial^29^, immunomodulatory^30^ and insecticidal^31^,^32^ activities. Several previous studies that performed chemical analysis of this plant reported the isolation and purification of various compounds including diterpenes, flavones, lignans and coumarins. Although various physiological activities have been reported, its effects on wound healing activity and its underlying mechanism have not been investigated.

This present study investigated the effect of *S. chamaejasme* extract (SCE) and its constituents on cutaneous wound healing *in vitro* and *in vivo*. The effects of SCE on the wound healing process were evaluated by measuring neoepidermis formation in the wounds. Furthermore, we assessed the effect of SCE and its constituents on cell migration and β-catenin activation in keratinocytes, collagen production in fibroblasts, and PGE_2_ inhibition in macrophages to confirm the role of SCE and its active compounds in wound healing in an *in vitro* system.

## Results

### Identification of seven compounds from S. chamaejasme

**Figure 1:**
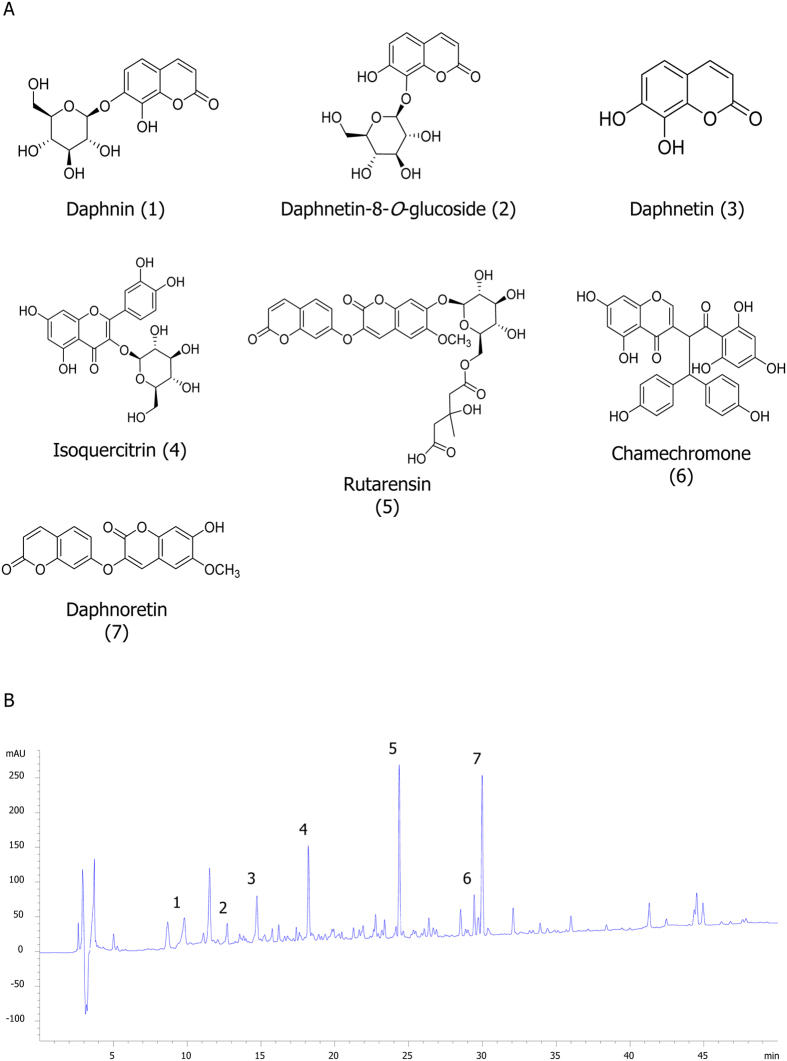
Chemical constituents isolated from *S. chamaejasme*. (**A**) Structure of compounds isolated from *S. chamaejasme*. (**B**) Chromatogram of SCE analyzed by HPLC detected at 330 nm and its assigned peaks; daphnin (peak 1), daphnetin-8-glucoside (peak 2), daphnetin (peak 3), isoquercitrin (peak 4), rutarensin (peak 5), chamechromone (peak 6), and daphnoretin (peak 7). The gradient profile was as follows: 0–5 min, initial mobile phase of 0.1% formic acid prepared in acetonitrile and 0.1% formic acid prepared in water (10:90); 5–45 min, linear gradient of 70:30; and 45–50 min, isocratic 70:30. The flow rate was 1.0 ml/min, and detection was at 254 nm. The sample concentration was 10 mg/min in MeOH, and the injection volume was 10 μl.

Seven compounds were identified from SCE using LC-NMR/MS: daphnin (compound 1, purity >98%), daphnetin-8-O-glucoside (compound 2, >97%), daphnetin (compound 3, >98%), rutarensin (compound 4, >98%), isoquercitrin (compound 5, >98%), chamechromone (compound 6, >98%), and daphnoretin (>98%). Compound purity was determined by HPLC (Fig. 1).

### SCE and its active compounds enhance the motility and differentiation of HaCaT cells by activating the β-catenin, ERK and Akt signalling pathways

**Figure 2:**
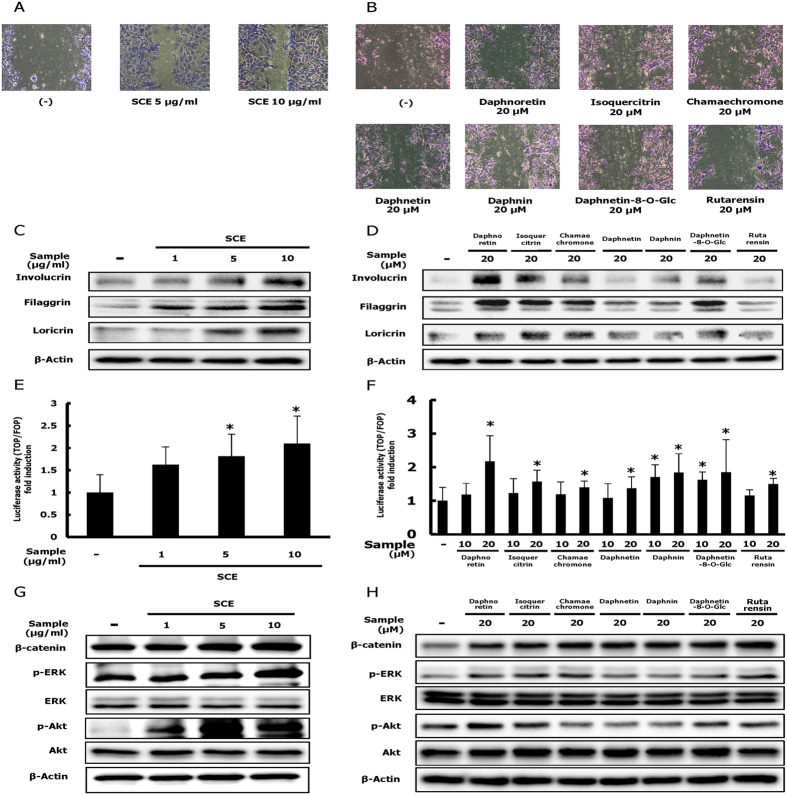
Effects of SCE and its constituents on HaCaT keratinocyte migration, differentiation, and β-catenin activation. (**A**,**B**) HaCaT keratinocytes were maintained in DMEM supplemented with 10% heat-inactivated FBS, streptomycin (100 mg/ml), and penicillin (100 mg/ml) in 5% CO_2_ at 37 °C. After scratch wounding with sterile pipette tips, HaCaT keratinocytes were incubated with medium containing 2% serum with or without SCE (1,5, or 10 μg/ml) or various compounds (20 μM) for 24 h. Cells were stained with crystal violet for 24 h (original magnification ×40). (**C**,**D**) Western blot analysis of filaggrin, loricrin, and involucrin in control and sample (SCE or various compounds)-treated HaCaT cells. (**E**,**F**) SCE (1,5, or 10 μg/ml) or various compounds (20 μM) were added to HaCaT cells for 24 h. Luciferase activity of HaCaT cells transfected with TOPflash/FOPflash was measured. (**G**,**H**) Western blot analysis of β-catenin, p-ERK, and p-Akt in control and sample (SCE or various compounds)-treated HaCaT cells. Results are expressed as the mean ± SD of three independent experiments. (^##^*p* < 0.01, ^*^p < 0.05 and ^**^p < 0.01).

To evaluate whether SCE and its constituents can influence keratinocyte migration and differentiation, we assessed the effect of SCE and its constituents on HaCaT cell motility and expression of keratinocyte differentiation markers. Treatment of HaCaT cell with SCE and its constituents for 24 hours increased the migration of keratinocytes (Fig. 2A and B). In addition, protein expression of filaggrin, loricrin and involucrin that are important keratinocyte differentiation markers, was increased by SCE and its active compounds (Fig. 2C and D).

Considering the correlation between wound healing and the β-catenin signalling pathway^3^, we investigated the effect of SCE and its constituents on β-catenin activation. SCE and its constituents increased nuclear translocation and protein expression of β-catenin (Fig. 2E–H). The ERK and Akt signalling pathways enhance keratinocyte motility^14^,^15^. Therefore, we assessed the effects of SCE and its constituents on phosphorylation of ERK and Akt. Treatment with SCE and its constituents increased the phosphorylation of ERK and Akt (Fig. 2G and H).

### SCE and its active compounds induce collagen expression in Hs68 cells

**Figure 3:**
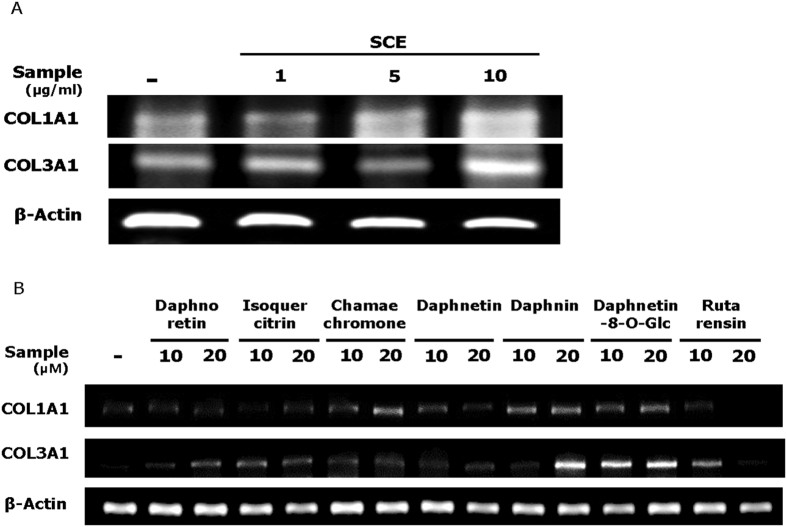
Effects of SCE and its constituents on mRNA expression of collagen types I and III (COL1A1, COL3A1) and production of type I procollagen. Human dermal fibroblasts (Hs68) were incubated for 48 h with SCE (1,5, or 10 μg/ml) or various compounds (10 or 20 μM). (**A**,**B**) mRNA levels of COL1A1, COL3A1 were determined with RT-PCR. (**C**,**D**) Production of type I procollagen was determined with ELISA. Results are expressed as the mean ± SD of three independent experiments. (^##^*p* < 0.01 and ^**^p < 0.01).

Fibroblast-to-myofibroblast transition plays a critical role in cutaneous wound healing^33^. mRNA levels of COL1A1 and COL3A1 and production of type І procollagen were markedly augmented by SCE and several compounds (especially daphnin and daphnetin-8-O-glucoside) in Hs68 cells, as shown by RT-PCR and ELISA analysis (Fig. 3A–D).

### SCE and chamechromone inhibit NO and PGE_2_ production and expression of inflammatory mediators in RAW 264.7 cells

**Figure 4:**
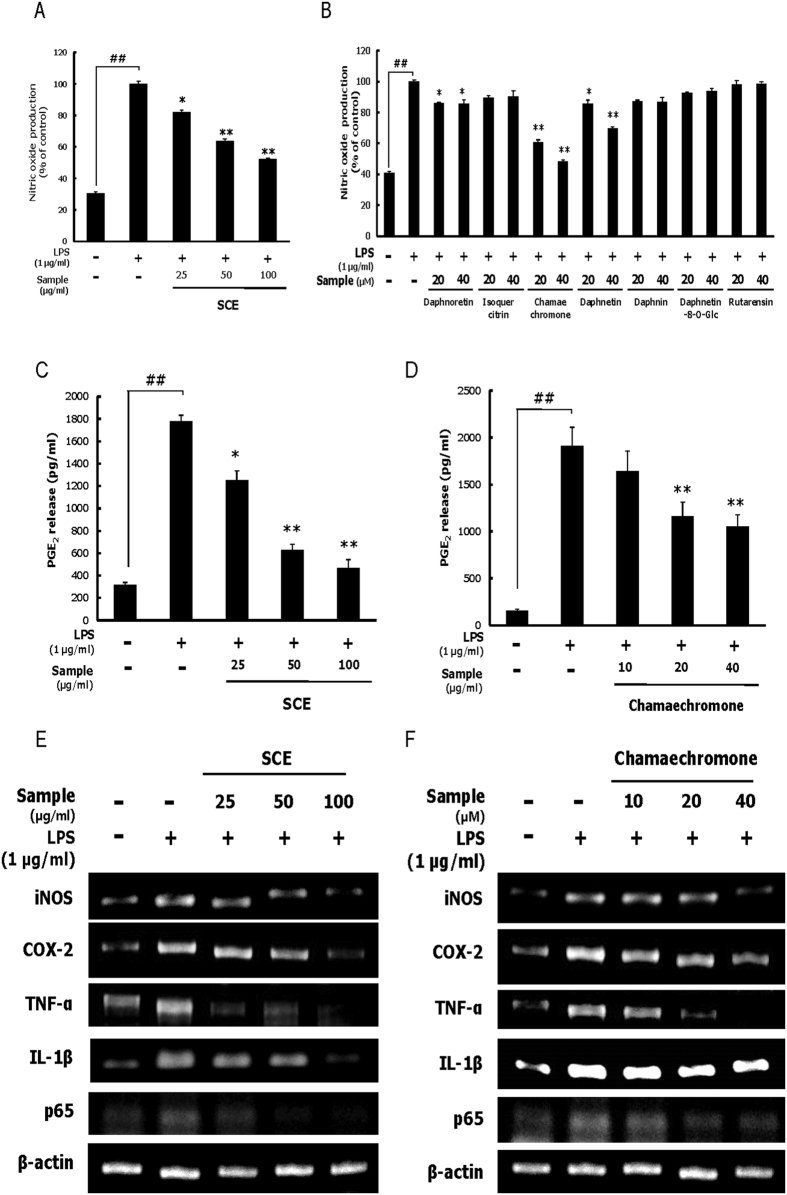
Effects of SCE and its constituents on the NO and PGE2 production and expression of inflammatory mediators in RAW 264.7 cells. RAW 264.7 cells were pretreated with different concentrations of SCE (25, 50, and 100 μg/ml) or various compounds (20 or 40 μM) for 1 h and then stimulated with LPS (1 μg/ml) for 24 h. (**A**,**B**) Nitrite levels were measured by the Griess reaction. (**C**,**D**) PGE_2_ levels were measured by the ELISA kit. (**E**,**F**) mRNA levels of inflammatory mediators were determined with RT-PCR. Results are expressed as the mean ± SD of three independent experiments. (^##^*p* < 0.01, ^*^p < 0.05 and ^**^p < 0.01).

The production of NO and PGE_2_ in LPS-stimulated RAW 264.7 cells was assayed after treatment with SCE or isolated compounds. SCE and chamechromone significantly suppressed LPS-induced NO production (Fig. 4A and B). In addition, SCE and chamechromone pretreatment reduced LPS-induced PGE_2_ production in a dose-dependent manner in RAW 264.7 cells (Fig. 4C and D). These data indicate that SCE and chamechromone significantly inhibit production of the inflammatory mediators NO and PGE_2_ in LPS-stimulated macrophages. Macrophages initiate an immune response by identifying and phagocytosing pathogens, inducing the secretion of inflammatory mediators such as iNOS, COX-2, TNF-α, IL-1β and p65^34^. Thus, we examined the expression of proinflammatory mediators that have a nuclear factor-κB-binding site in their promoter region^35^. To investigate the effects of SCE and chamechromone on the LPS-induced mRNA expression of proinflammatory cytokines, the mRNA levels of iNOS, COX-2, TNF-α, IL-1β and p65 in RAW 264.7 cells were measured using RT-PCR. Treatment with SCE and chamechromone significantly inhibited the production of iNOS, COX-2, TNF-α, IL-1β and p65 (Fig. 4E and F). These results suggest that SCE and chamechromone suppress the LPS-induced expression of proinflammatory cytokines.

### SCE promotes cutaneous wound healing

**Figure 5:**
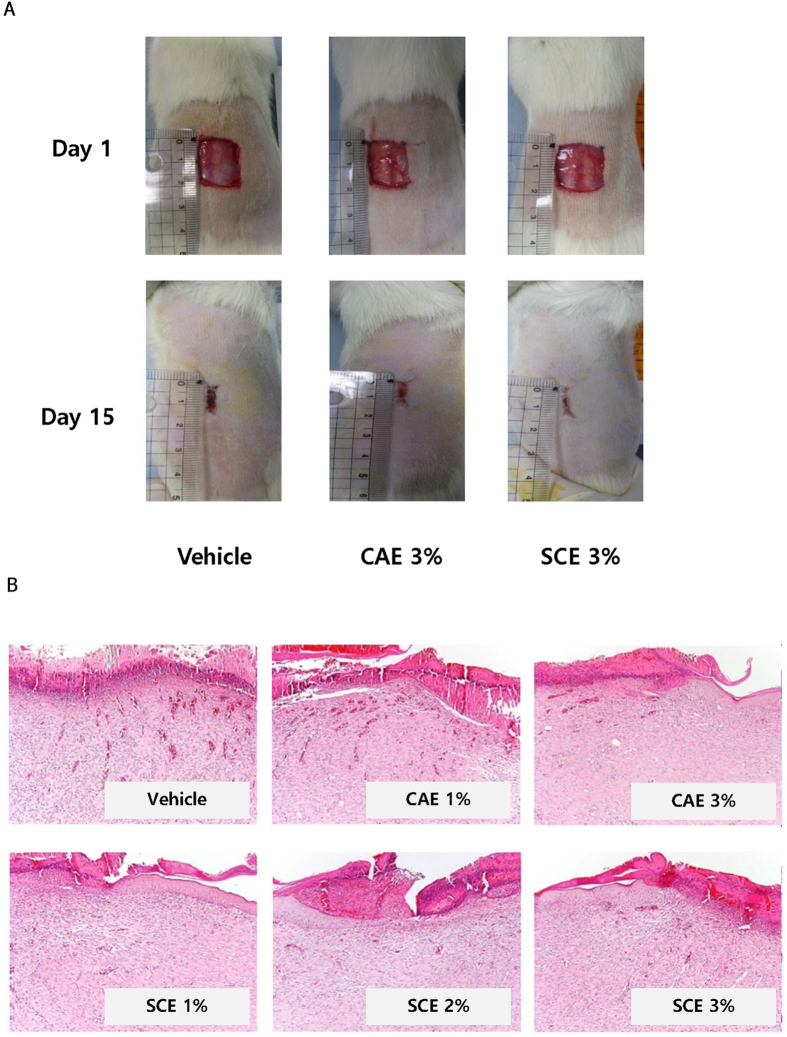
Effect of SCE on cutaneous wound healing. A full-thickness skin excision (diameter = 1.5 cm) was made on the backs of 8-week-old SD rat, and SCE (1, 2, or 3%) or *Centella asiatica* extract (CAE, 1 or 3%) was topically applied to the wounds daily. Tissues were excised from the wounded area and fixed in paraformaldehyde for immunohistochemistry. (**A**) Representative gross images of wounded skin treated by SCE or CAE for 15 day. (**B**) Representative H&E stained tissues of wounded skin treated with or without SCE or CAE (original magnification ×100).

We assessed the effect of SCE on cutaneous wound healing. After topical application of SCE (1%, 2% or 3%) to the wound areas of SD rats, SCE significantly reduced the wound size and re-epithelialised skin lesions (Fig. 5A, Tables 1 and 2). Histological analysis (H&E staining) also revealed restoration of normal tissue structure after SCE treatment (Fig. 5B).

## Discussion

The proliferative phase of wound healing is associated with the re-epithelialisation process including collagen production and ECM remodelling^36^. During wound re-epithelialisation stage, skin lesions closes the wound and remodels the cytoskeleton by enhancing the proliferation and migration of keratinocytes^37^. In this study, SCE and its constituents augmented the expression of keratinocyte differentiation markers, which are influenced by the β-catenin signalling pathway^38^, in HaCaT keratinocytes. The β-catenin signalling pathway enhances the proliferation and motility of keratinocytes^10^,^11^. Although dysregulation of the β-catenin signalling pathway induces hypertrophic keloids and scars^39^, adequate activation of this pathway in wounds is crucial to improve wound healing. It was reported that the proliferation of keratinocytes is regulated by inhibition of apoptosis during early wound healing^10^. Our results indicate that SCE and its constituents enhance the proliferation of keratinocytes by activating ERK and Akt signalling.

During the early stage of wound healing, collagen production is promoted to increase scar formation, and then it is gradually decreased to normal levels in the final stage of wound healing^40^. Several studies verified that TGF-β1 plays a significant role in the re-epithelisation process by increasing ECM production and inhibiting inflammation^41^. Furthermore, the TGF-β1 signalling pathway also interacts with the β-catenin signalling pathway during the wound healing process^42^,^43^. It was reported that Wnt3a treatment increases the levels of differentiation markers in myofibroblasts by activating the TGF-β1 signalling pathway^44^. Our results suggest that SCE and its constituents increased the expression of COL1A1, COL3A1 and TGF-β1 in dermal fibroblasts by activating the β-catenin signalling pathway.

Many bioactive mediators, such as eicosanoids, cytokines and growth factors, regulate each stage of the wound healing process. During infection and inflammation, activated macrophages that have infiltrated the site of inflammation produce a large amount of NO around the wounded tissues^45^. NO is a highly reactive radical that is generated by the activation of iNOS and contributes to various biological processes including inflammation. NO is thought to be a key disruptive factor in the wound healing process^46^, although it was reported that small amounts of NO may enhance wound healing during the early stage^47^. Excessive generation of PGE_2_ by COX-2 is a crucial physiological factor accelerating inflammation^48^. PGE_2_ is related to keratinocyte proliferation^49^, angiogenesis^50^ and mediation of the inflammatory response^51^. Our results suggest that SCE and chamechromone inhibit LPS-induced NO and PGE_2_ generation through inhibition of iNOS and COX-2 mRNA expression in RAW 264.7 macrophages. However, further investigation of the underlying mechanism is necessary. Macrophages are key factors in inflammation and reduce various harmful stimuli. Furthermore, they trigger the LPS-induced inflammatory response by producing proinflammatory mediators such as TNF-α, IL-1β and p65^52^. Overproduction of these mediators leads to excessive inflammatory responses^53^. Therefore, suppression of proinflammatory mediator production may be helpful in reducing the inflammatory response. In this study, SCE and chamechromone suppressed production of TNF-α, IL-1β and p65 in RAW 264.7 macrophages stimulated by LPS. Our results demonstrate that SCE and chamechromone suppress production of inflammatory mediators in RAW 264.7 cells.

We found that SCE promoted the epithelialization and wound closure rate in SD rat model. CAE, which is a famous traditional medicines proven to be effective in wound healing treatment^54^,^55^, was used as a positive control. Based on various studies, the biologically active constituents of CAE are known to be madecassic acid, asiatic acid, madecassoside and asiaticoside^56^,^57^. In our study, SCE group (SCE 3%) showed the enhanced cutaneous wound healing activity compared with CAE group (CAE 3%).

According to our data, all of eight tested constituents of SCE play a role at different molecular targets for exerting various biological effects in the wound healing system. For example, most of the constituents increase the keratinocyte migration, proliferation, and differentiation and β-catenin activation in HaCaT keratinocytes. In addition, daphnin and daphnetin-8-O-glucoside augmented collagen production and expression in Hs68 fibroblasts. Chamechromone inhibited the production of inflammatory mediators in Raw 264.7 macrophages. Therefore, in addition to the results that shows the positive effects of SCE on overall stages of wound healing, our results indicate that individual constituents of SCE exert their beneficial activity on different stages of wound healing process by regulating inflammatory, proliferative and remodelling phases.

In conclusion, our results suggest that SCE and its constituents induce cutaneous wound healing in SD rats, enhance keratinocyte motility and mRNA expression of type I and III collagen and inhibit PGE_2_ production and inflammatory mediator expression. SCE enhanced re-epithelialisation in wounds at an early stage of wound healing. Many wound healing drugs, including chemical molecules and growth factors, often have serious side effects^58^. However, SCE can be used to treat cutaneous wounds without any side effects. Therefore, SCE and its constituents may be beneficial for the treatment of wounds.

## Materials and Methods

### Reagents and materials

Foetal bovine serum (FBS), penicillin and Dulbecco’s modified Eagle medium (DMEM) were purchased from Hyclone (South Logan, UT, USA). Antibodies targeting β-catenin, phospho-p44/42 MAPK (ERK1/2), p44/42 MAPK (ERK1/2), phospho-Akt (Tyr525/526), Akt, phospho-Smad2 (Ser465/467)/Smad3 (Ser423/425), Smad2/3, and β-actin were purchased from Cell Signaling Technology Inc. (Danvers, MA, USA). Antibodies targeting filaggrin, loricrin, and involucrin were purchased from Santa Cruz Biotechnology (Santa Cruz, CA, USA). LPS (*Escherichia coli*, serotype 0111:B4) and all other chemicals were purchased from Sigma Chemical (St. Louis, MO, USA). The plasmid (β-catenin) was transfected using Lipofectamine Plus (Invitrogen, Carlsbad, CA, USA) according to the manufacturer’s guidelines. *Centella asiatica* extract (CAE), as a positive control, was obtained from Korea Chong Kun Dang Pharma. Co. Ltd., in Korea.

### Plant material

*S. chamaejasme* was collected in Gachuurt, Ulaanbaatar, Mongolia and was identified by Dr C. Sanchir, Institute of Botany, Mongolian Academy of Sciences. A voucher specimen was placed in the Flora and Plant Systematic Laboratory, Institute of Botany, Mongolian Academy of Sciences.

### Extraction and isolation of compounds

Dried aerial parts of *S. chamaejasme* plants (5 kg) were extracted with 95% ethanol for 10 days at room temperature and filtered through filter paper. The filtrates were evaporated in a vacuum to yield the ethanol extract (203 g). This ethanol extract was suspended in distilled water and partitioned with *n*-hexane, ethyl acetate and *n*-butanol. The *n*-butanol fraction (5.2 g) was chromatographed on a RP-18 column, eluting with a H_2_O-MeOH gradient system (60:40 → 50:50), to give five fractions (fractions 1–5). Compound **1** (daphnin) was purified from fraction 3 (980 mg) using Sephadex LH-20 (MeOH) and Silica gel [CH_2_Cl_2_-MeOH-Water (3:1:0.1 → 1:1:0.1)] column chromatography. From fraction 5 (200 mg), compound **2** (daphnetin-8-O-glucoside) was isolated with Sephadex LH-20 (MeOH) column chromatography.

The ethyl acetate fraction (22 g) was chromatographed on Silica gel using CH_2_Cl_2_-MeOH (50:1 → 0:100) to give 13 fractions (fractions 1–13). From fraction 4 (1.2 g), compound **3** (daphnetin) was re-chromatographed on a RP-18 column, eluting with a H_2_O-MeOH gradient system (60:40 → 50:50) and Sephadex LH-20 (MeOH). Compounds **4** (rutarensin) and **5** (isoquercitrin) were purified from fraction 13 (2.3 g) using Silica gel [CH_2_Cl_2_-MeOH-Water (5:1:0.1 → 1:1:0.1)] and Sephadex LH-20 (MeOH) column chromatography. From fraction 8 (240 mg), compound **6** (chamaechromone) was re-chromatographed on a RP-18 column, eluting with a H_2_O-MeOH gradient system (50:50 isocratic) and Silica gel [CH_2_Cl_2_-MeOH-Water (8:1:0.1 → 4:1:0.1)]. Compound **7** (daphnoretin) was isolated from fraction 3 on Silica gel, eluting with Hexane-EtOAc (2:1 → 1:1), and recrystallised with CHCl_3_-MeOH to obtain white crystals.

The chemical structures of compounds 1–7 (Fig. 1A) were determined by ^1^H and ^13^C nuclear magnetic resonance data and compared with reported data^59^,^60^,^61^,^62^,^63^,^64^,^65^.

### High-performance liquid chromatography (HPLC) analysis

The high-performance liquid chromatography (HPLC) system consisted of an Agilent infinity series 1260 liquid chromatography system with a G1311B quaternary pump, G1329B autosampler, G1316A column oven and G1315D DAD detector, connected to Agilent ChemStation software (Agilent, Waldbronn, Germany). The separation was conducted with a YMC-PACK ODS column (4.6 mm × 250 mm, 5 μm). The gradient profile was as follows: 0–5 min, initial mobile phase of 0.1% formic acid prepared in acetonitrile and 0.1% formic acid prepared in water (10:90); 5–45 min, linear gradient of 70:30; and 45–50 min, isocratic 70:30. The flow rate was 1.0 ml/min, and detection was at 254 nm. The sample concentration was 10 mg/min in MeOH, and the injection volume was 10 μl. The HPLC chromatogram of SCE is shown in Fig. 1B.

### Cell culture

The human keratinocyte cell line (HaCaT), human foreskin fibroblast cell line (Hs68) and mouse macrophage cell line (RAW 264.7) were obtained from the American Type Culture Collection (Manassas, VA, USA) via the Korean Cell Line Bank (Seoul, Korea), and were routinely cultured in DMEM supplemented with 10% FBS and 1% penicillin-streptomycin. Cells were grown in a humidified atmosphere with 5% CO_2_ at 37 °C.

### Cell viability assay

Cell viability was assayed using a 3-[4,5-dimethylthiazol-2-yl]-2,5-diphenyltetrazolium bromide (MTT) colorimetric assay (Sigma-Aldrich, St Louis, MO, USA). HaCaT, Hs68 and RAW 264.7 cells were grown in 24-well plates (1 × 10^5^ cells/ml) for 24 h. After treatment with various concentrations of SCE and its constituents for 6 h, cells were washed and incubated with MTT (0.5 mg/ml) at 37 °C for 4 h. Cells were then washed, and the insoluble formazan products were dissolved in 200 μl of DMSO. Absorbance was measured at 550 nm by spectrophotometry (Bio-Tek Instruments, Winooski, VT, USA).

### Cell migration assay

HaCaT cells were seeded into 24-well plates (1 × 10^5^ cells/well). After 24 h of incubation, the monolayers were scratched with sterile pipette tips and treated with or without SCE (5 or 10 μg/ml) and its constituents (20 μM). After 48 h, the cells were fixed in 4% formalin for 20 min and stained with 2% crystal violet. The wound closure rate was evaluated using an Eclipse TE2000U inverted microscope with twin CCD cameras (magnification, ×200; Nikon, Tokyo, Japan; n = 3).

### Reporter assay

Luciferase transactivation was measured as described previously^66^. TOPflash and FOPflash assays were performed in HaCaT cells. Cells were seeded in 24-well plates (6 × 10^4^ cells/well). After 24 h, cells were co-transfected with luciferase reporter constructs (TOPflash or FOPFlash) and the pRL-CMV-Renilla reporter plasmid using Fugene 6 (Roche Applied Science, Indianapolis, IN, USA). After 24 h, SCE (5 or 10 μg/ml) and its constituents (10 or 20 μM) were added to the HaCaT cells for another 24 h. The cells were then lysed with lysis reagent (Promega, Madison, WI, USA), and the luciferase assay substrate (Promega) was added using a dual-luciferase assay system according to the manufacturer’s instructions. Transcriptional activity values were measured using a luminometer (Bio-Tek Instruments).

### Enzyme-linked immunosorbent assay (ELISA) and NO assay

RAW 264.7 cells were incubated with SCE (50, 100 or 200 μg/ml) and its constituents (20 or 40 μM) for 1 h and stimulated with LPS (1 μg/ml) for 24 h. Cell culture medium was collected, and the secretion of PGE_2_ was determined using enzyme-linked immunosorbent assay (ELISA) kits (R&D Systems, Minneapolis, MN, USA) according to the manufacturer’s instructions. Nitrite (indicator of NO production) was measured in culture medium by adding Griess reagent. The culture medium was mixed with Griess reagent for 20 min, and then the absorbance at 540 nm was measured by spectrophotometry (Bio-Tek Instruments).

Hs68 cells were incubated with serum-free DMEM containing SCE (1, 5 or 10 μg/ml) and its constituents (20 μM). Cell culture medium was collected after 24 h, type I procollagen production were quantified using a procollagen type I C-peptide enzyme immunoassay kit (MK101; Takara, Shiga, Japan).

### Animal study

Seven-week-old male Sprague-Dawley (SD) rats (Central Laboratory Animal Inc., Seoul, Korea) were housed in a controlled environment (25 ± 2 °C, 55% ± 5% relative humidity, 12 h light-dark cycle) and allowed to acclimatise for 1 week. All mouse experiments were performed in accordance with the Guide for the Care and Use of Laboratory Animals and were approved by the Institutional Animal Care and Use Committee of the Korea Institute of Science and Technology in Seoul, Korea (2015–017). One day after dorsal hair removal, full-thickness incision wounds were made. SCE (1%, 2% or 3%) was topically applied to the wounds every day (n = 10), and wound sizes were measured three times per week. At the end of experiments, animals were anaesthetised with a Zoletil-Rompun mixture and skin lesions were removed and stored at −80 °C until analysis.

### Histological analysis

Skin lesions were embedded in paraffin, sectioned (4 mm thick), deparaffinised in xylene and rehydrated in a gradient of alcohol solutions. The sections were stained with haematoxylin-eosin (H&E). The H&E-stained slides were visualised using an Eclipse TE2000U inverted microscope with twin CCD cameras (magnification, ×200; Nikon).

Abramov’s histological scoring system was used to score epithelisation, fibrosis, angiogenesis and collagen levels^67^,^68^. This system assigns a score of 0–3 for each parameter. Inflammation (the number of macrophages) was scored as follows: 0–25 = 1, 26–50 = 2 and >51 = 3. Fibroplasia was graded as follows: 0 (none to minimal fibroblasts), 1 (a few fibroblasts), 2 (more fibroblasts) and 3 (predominantly fibroblasts). Angiogenesis was graded as follows: 0 (none), 1 (up to five vessels per high-power field [HPF]), 2 (6–10 vessels per HPF) and 3 (more than ten vessels per HPF). Epithelisation was graded as follows: 0 (none), 1 (partial), 2 (complete, but immature or thin) and 3 (complete and mature).

### Reverse transcription-polymerase chain reaction (RT-PCR)

Total RNA was isolated from the skin lesion using TRIzol reagent (Invitrogen) according to the manufacturer’s instructions and quantified by spectrophotometry at 260 nm. An equal amount of total RNA was used to synthesise cDNA with reverse transcriptase premix (Elpis-Biotech. Inc., Taejeon, Korea). Reverse transcription was performed at 42 °C for 55 min and was terminated by incubation at 94 °C for 5 min. PCR amplification of the cDNA products (3 μl) was performed with PCR premix (Elpis-Biotech) and the primer pairs (Bioneer, Daejeon, Korea) shown in Table 1. Prior to PCR amplification, primers were denatured at 94 °C for 5 min. PCR was performed on a GeneAmp PCR System 2700 (Applied Biosystems, Foster City, CA, USA). PCR products were separated by 1.5% agarose gel electrophoresis and visualised with ethidium bromide. β-Actin was used as an internal control for normalising data.

### Western blot analysis

HaCaT and Hs68 cells were lysed in RIPA buffer containing protease inhibitors and then incubated on ice for 10 min. The protein concentrations of the lysates were normalised by the Bradford method (Bio-Rad Laboratories, Hercules, CA, USA). Equal amounts of protein (20 μg) were resolved by 10% SDS-polyacrylamide gel electrophoresis and transferred to nitrocellulose membranes (Whatman GmbH, Dassel, Germany). The membrane was further incubated with specific primary antibodies for 16 h at 4 °C, followed by appropriate secondary antibodies coupled to horseradish peroxidase (Santa Cruz Biotechnology, Santa Cruz, CA, USA) for 2 h. Proteins were developed with ECL Western detection reagents (Amersham Biosciences, Little Chalfont, UK) and visualised by enhanced chemiluminescence (GE Healthcare, Hatfield, UK).

### Statistical analysis

Each experiment was performed at least three times. All data are expressed as mean ± standard deviation. Differences between groups were analysed using a one-way analysis of variance followed by Scheffe’s test (SPSS 17.0, Chicago, IL, USA). ^##^*p* < 0.01, ^*^p < 0.05 and ^**^p < 0.01 were considered statistically significant.

## Additional Information

**How to cite this article:** Kim, M. *et al*. *Stellera chamaejasme* and its constituents induce cutaneous wound healing and anti-inflammatory activities. *Sci. Rep.* **7**, 42490; doi: 10.1038/srep42490 (2017).

**Publisher's note:** Springer Nature remains neutral with regard to jurisdictional claims in published maps and institutional affiliations.

## Figures and Tables

**Table 1:** Effect of SCE on histological score in SD rat.

Histological score	Vehicle	CAE 1%	CAE 3%	SCE 1%	SCE 2%	SCE 3%
Inflammation	2.5 ± 0.3	2.3 ± 0.3	2.3 ± 0.3	2.3 ± 0.3	2.2 ± 0.2	2.3 ± 0.3
Fibroplasia	1.7 ± 0.3	1.8 ± 0.2	1.9 ± 0.1	2 ± 0.1	2.1 ± 0.1	2.2 ± 0.2*
Angiogenesis	2.5 ± 0.3	2.7 ± 0.3	2.2 ± 0.2	2.4 ± 0.3	2.3 ± 0.3	2.3 ± 0.3
Epithelization	0.3 ± 0.2	0.6 ± 0.3	0.8 ± 0.2*	0.9 ± 0.2*	0.8 ± 0.1*	1.0 ± 0.2**

Results are expressed as mean ± S.D. (% control). (^##^*P* < 0.01 compared with control group; ^*^P < 0.05, ^**^P < 0.01 compared with Vehicle group. n = 10/group).

**Table 2:** Effect of SCE on cutaneous wound area in SD rat.

Wound area (cm^2^)	Vehicle	CAE 1%	CAE 3%	SCE 1%	SCE 2%	SCE 3%
Day 1	5.66 ± 0.74	5.77 ± 0.91	5.93 ± 0.77	5.47 ± 0.51	5.25 ± 0.56	5.65 ± 0.44
Day 4	5.73 ± 0.55	4.89 ± 0.75	5.04 ± 0.73	5.32 ± 0.46	4.93 ± 0.4	4.76 ± 0.78
Day 8	3.81 ± 0.55	3.46 ± 0.46	3.1 ± 0.51	3.08 ± 0.39	2.95 ± 0.49	2.12 ± 0.4**
Day 11	2.25 ± 0.49	1.99 ± 0.28	1.66 ± 0.14	1.53 ± 0.14*	1.73 ± 0.34	1.07 ± 0.22**
Day 15	0.97 ± 0.14	0.9 ± 0.21	0.71 ± 0.08*	0.67 ± 0.06*	0.62 ± 0.15*	0.47 ± 0.07**

Results are expressed as mean ± S.D. (% control). (^##^*P* < 0.01 compared with control group; ^*^P < 0.05, ^**^P < 0.01 compared with Vehicle group. n = 10/group).
